# The PICASSO Cohort: baseline characteristics of a cohort of men who have sex with men and male-to-female transgender women at high risk for syphilis infection in Lima, Peru

**DOI:** 10.1186/s12879-017-2332-x

**Published:** 2017-04-11

**Authors:** Noah Kojima, Hayoung Park, Kelika A. Konda, Dvora L. Joseph Davey, Claire C. Bristow, Brandon Brown, Segundo R. Leon, Silver K. Vargas, Gino M. Calvo, Carlos F. Caceres, Jeffrey D. Klausner

**Affiliations:** 1 David Geffen School of Medicine, University of California Los Angeles, 10833 Le Conte Ave, Los Angeles, CA 90095 USA; 2 Unit of Health, Sexuality and Human Development and Laboratory of Sexual Health, Universidad Peruana Cayetano Heredia, Lima, Peru; 3 Department of Epidemiology, School of Public Health, University of California Los Angeles, Los Angeles, CA USA; 4 Division of Global Public Health, Department of Medicine, University of California San Diego, La Jolla, CA USA; 5 Center for Healthy Communities, University of California Riverside, Riverside, CA USA; 6 Epicentro Salud, Lima, Peru

**Keywords:** Syphilis, Treponema Pallidum, Human immunodeficiency virus, HIV, Sexually transmitted infection, STI, Men who have sex with men, MSM, Transwomen, Peru

## Abstract

**Background:**

Men who have sex with men (MSM) and male-to-female transgender women (transwomen) are disproportionately at risk of syphilis infection in Peru.

**Methods:**

From 2013 to 2014, MSM and transwomen seeking human immunodeficiency virus (HIV) or sexually transmitted infection (STI) testing and/or treatment were recruited into a 2-year observational cohort study to determine predictors of recently acquired syphilis infection (defined as a rapid plasma reagin [RPR] titer ≥1:16 and a reactive treponemal antibody test) in Lima, Peru. At baseline, interviewers collected sociodemographic, behavioral, and medical characteristics from participants. All cohort participants were tested for syphilis, HIV, *Chlamydia trachomatis* (CT), and *Neisseria gonorrhoeae* (NG) infection. Using cross-sectional analyses, bivariate and multivariate models were used to determine factors associated with recently acquired syphilis infection and calculate adjusted prevalence ratios.

**Results:**

We recruited 401 participants, 312 MSM and 89 transwomen, with median ages of 29.0 and 32.5 years old (interquartile ranges: 23.3, 37.4 and 27.2, 39.5, respectively). The prevalence of recently acquired syphilis infection at baseline was 16.8% for MSM and 6.7% for transwomen. Among MSM and transwomen, 30.1 and 33.7% were infected with HIV, 18.6 and 24.7% were infected with CT, and 14.2 and 19.1% were infected with NG, respectively. Co-infection rates among MSM with recently acquired syphilis infection included: 44.2% with HIV, 40.4% with CT (32.7% with anal CT and 7.7% with pharyngeal CT), and 19.2% with NG (11.5% with anal NG and 7.7% with pharyngeal NG). Co-infection rates among transwomen with recently acquired syphilis infection included: 66.7% with HIV, 0% with CT, and 16.7% with anal NG. In multivariate analysis among the entire cohort, recently acquired syphilis infection was independently associated with younger age (adjusted prevalence ratio [aPR] = 0.96, 95% confidence interval [CI] = 0.93–0.99), receptive role during anal sex (aPR = 2.56, 95% CI = 1.05–6.25), prior HIV diagnosis (aPR = 1.70, 95% CI = 1.11–2.61), anal CT or NG infection (aPR = 1.69, 95% CI = 1.09–2.60), and prior syphilis diagnosis (aPR = 3.53, 95% CI = 2.20–5.68).

**Conclusions:**

We recruited a cohort of MSM and transwomen who had a high prevalence of recently acquired syphilis infection in Lima, Peru. Recently acquired syphilis infection was associated with socio-demographic characteristics, sexual risk, and sexually transmitted co-infections.

## Background

Despite syphilis being a curable sexually transmitted infection (STI) with proven diagnostics and known treatment and prevention strategies, it is estimated that 6 million incident syphilis cases occur worldwide annually [1]. In South America and Peru, research indicates that syphilis is hyperendemic among men who have sex with men (MSM) and male-to-female transgender women (transwomen) [2–4]. In 2008, the prevalence of syphilis infection, verified by laboratory testing, was estimated to be 28.9% among MSM in Lima, Peru [5].

As evidenced by persistent incident syphilis infection among MSM and transwomen in Peru, current screening and management strategies are inadequate [6]. Syphilis is associated with increased risk of HIV infection, therefore quality prevention and treatment measures could have the added benefit of mitigating HIV acquisition and transmission [7]. Indeed, more research is needed to characterize determinants of syphilis among groups who are at highest risk. To better address ongoing endemic infections, these data will be presented to the Ministry of Health of Peru with recommendations to guide HIV and syphilis infection control strategies in Peru [8]. In this manuscript, we report baseline characteristics and behaviors associated with incidence of recently acquired syphilis infection using quality laboratory testing and results from socio-demographic and behavioral surveys among a clinic-based observational cohort of MSM and transwomen in Lima, Peru.

## Methods

### Study population and consent to publish

Participants were recruited at two clinics that serve MSM and transwomen—Epicentro, located in the Barranco district, and Alberto Barton Health Center, located in the Callao district. Epicentro is a community-based non-governmental organization for MSM and transwomen that provides HIV/STI testing, medical care, and community activities. The Alberto Barton Health Center is a government run HIV and STI clinic that provides testing and medical care specifically for MSM and transwomen.

To recruit a study cohort of MSM and transwomen who were at increased risk of syphilis infection, we developed inclusion criteria by adapting risk score protocols from other scientific fields used for identification of suitable participants [8, 9]. Eligible participants were: MSM or transwomen, aged ≥18 years, seeking testing and/or care for human immunodeficiency virus (HIV) or an STI, willing to return for follow-up appointments every 3 months over 2 years (eight follow-up appointments in total), and have at least three of the following traits: (i) a positive syphilis rapid treponemal test; (ii) a reactive HIV rapid test; (iii) 5 or more years of sexual activity; (iv) five or more sex partners in the past 3 months; (v) diagnosis of an STI in the last 6 months; (vi) genital ulceration; or (vii) five or more episodes of condomless anal intercourse in the past 6 months.

Study procedures, risks and benefits of participating in the study, the option to discontinue study participation at any time, and a means to contact investigators and study coordinators were provided to participants. Written informed consent was obtained prior to study initiation. Participants were reimbursed approximately $5 in local currency for transportation. Condoms, lubricants, and all study related testing and clinical care were provided at no cost to participants.

### Study procedures

Demographic, gender, and health and sexual behavior data were collected by trained study staff using computer-assisted structured interviews. Demographic characteristics included: date of birth, area of residence, education, income, and living arrangement. Gender was self-reported. Health history and behavior included: any past syphilis infections, antibiotic use, and knowledge of HIV. Locations of recent (in the past three months) sexual encounters, use of alcohol or drugs during sex, payment for sex, gender of sex partners, number of sex partners, types of relationships with sex partners, and role during sex were also recorded. Role during anal sex was defined as insertive, receptive, or versatile (both insertive and receptive). Surveys assessed whether participants had received a prior diagnosis of HIV infection by a health care provider and if participants with a diagnosis of HIV infection were using antiretroviral therapy (ART). Alcohol use was assessed using the WHO Alcohol Use Disorders Identification Test and scores were dichotomized with ≥8 indicating an alcohol use disorder [10].

A trained health care provider examined participants after they completed the survey. Participants provided biological samples for diagnostic testing.

### Laboratory testing

Rapid point-of-care tests were used to assess participants for syphilis (Alere Determine™ Syphilis TP, Alere Inc., Waltham, MA, USA) and HIV infection (Alere Determine™ HIV 1/2, Alere Inc., Waltham, MA, USA). All participants were assessed for syphilis with rapid plasma reagin (RPR) (BD Macro-Vue™ RPR Card Test Kit, Beckton Dickinson, Franklin Lakes, NJ) and Treponema Pallidum Particle Agglutination (TPPA) tests (Serodia, Fujirebio Diagnostics Inc., Tokyo, Japan) using a cutoff value of ≥1:80. The highest reactive RPR titer was reported and recorded. Recently acquired syphilis infection was defined as a RPR titer ≥1:16 and a positive TPPA test. Prior syphilis was defined as RPR titers from 1:1–1:8 and a positive TPPA test. All participants were tested with HIV Ag/Ab EIA 4th generation sera tests (Genscreen ULTRA HIV Ag-Ab, Bio-Rad, Hercules, CA). Confirmation Western Blot tests were conducted (NEW LAV BLOT I, Bio-Rad, France) on all specimens with positive rapid HIV testing results. Participants with confirmed HIV infection were referred to the Peruvian National HIV Treatment Program [6].

Confirmation testing for syphilis and HIV infection, as well as testing for *Neisseria gonorrhoeae* (NG) and *Chlamydia trachomatis* (CT) infections, was performed at the Universidad Peruana Cayetano Heredia Sexual Health Laboratory. Clinician-collected pharyngeal specimens and self-collected anal swabs were tested for CT and NG infection using a transcription mediated amplification assay (Aptima Combo 2 Assay, Hologic, Inc., San Diego, USA). Urethral testing for STIs was not conducted in this study because the prevalence of asymptomatic urethral STIs in this population was very low in prior studies [11, 12].

Rapid test results were available on the day of testing. All other test results were delivered 2 weeks post-visit. Patients received appropriate post-test counseling, treatment (when indicated), and were requested to inform their sex partners about any STI, following Peruvian Ministry of Health protocol [6]. STI treatment was offered on-site according to Centers for Disease Control and Prevention and Peruvian national guidelines [13].

### Statistical analyses

StataSE 14.1 (College Station, TX) was used to perform descriptive statistics for inclusion criteria, characteristics of the study cohort, and prevalence of infection of the cross-sectional data for MSM and transwomen. Latent class analysis was used to assess whether any identifiable subgroups were present in the cohort [14]. Bivariate and multivariate analyses explored associations with recently acquired syphilis infection within the total cohort. To increase the sample size, we did not separate MSM and transwomen in those analyses. Individuals with recently acquired syphilis infection were compared to participants with non-reactive RPR tests. Participants with laboratory testing indicating prior syphilis (RPR titers from 1:1–1:8) were excluded from regression analyses. Characteristics of participants with recently acquired syphilis infection were compared to characteristics of participants with no history of syphilis using Poisson regression to compute prevalence ratios with 95% confidence intervals (CI). The multivariate model included all statistically significant (*p*-value of <0.05) variables in the bivariate analysis.

### Ethics, consent, and permissions

The Institutional Review Board at Universidad Peruana Cayetano Heredia and Alberto Barton Health Center provided ethical approval and oversight of the study under reference number SIDISI 59996. The University of California Los Angeles institutional review board determined that analysis of de-identified data was exempt from human subjects' considerations.

## Results

From May 2013 to May 2014, 401 participants, 312 MSM and 89 transwomen, were recruited into the PICASSO cohort in Lima, Peru. The median ages of MSM and transwomen were 29.0 and 32.5 years (interquartile ranges [IQR]: 23.3, 37.4 and 27.2, 39.5, respectively). Among participants, 27.9% of MSM and 76.5% of transwomen earned less than minimum wage (approximately $250 USD per month; Table 1). In survey results collected from participants prior to laboratory testing, 35.6% of MSM and 39.3% of transwomen reported a prior diagnosis of syphilis and 24.1% of MSM and 27.6% of transwomen reported a prior diagnosis of HIV infection. Among participants with a self-reported prior diagnosis of HIV infection, 54.8% of MSM and 52.4% of transwomen reported that they were not using ART (Table 1). MSM reported that during anal sex, 50.3% were a versatile partner (i.e. having receptive and insertive anal sex), 21.8% were a receptive partner, and 27.9% were an insertive partner. Transwomen reported that during anal sex, 30.3% were a versatile partner, 67.4% were a receptive partner, and 2.3% were an insertive partner. The median number of recent male or transwomen sex partners reported by MSM and transwomen was 4 (IQR: 2, 7) and 15 (IQR: 5, 40), respectively.

**Table 1:** Baseline characteristics of men who have sex with men and male-to-female transgender participants of the PICASSO study, Lima, Peru, 2013–2014

Characteristic	MSM	Transwomen
	n (*N* = 312) (%)	n (*N* = 89) (%)
*Survey*		
Age median years (Interquartile range [IQR])	29.0 (23.3, 37.4)	32.5 (27.2, 39.5)
Income less than living wage^a^	87 (27.9)	62 (76.5)
Education		
Primary school or less	7 (2.2)	8 (9.0)
Secondary school or less	111 (35.6)	65 (73.0)
Vocational school graduate	69 (22.1)	11 (12.4)
University student	90 (28.9)	3 (3.4)
University graduate or higher	40 (11.2)	2 (2.3)
Work		
Full time	157 (50.3)	33 (37.1)
Part time	41 (13.1)	23 (25.8)
Odd jobs	46 (14.7)	26 (29.2)
Unemployed or does not work	68 (21.8)	7 (7.9)
Use of antibiotics in the last 3 months	123 (39.4)	37 (41.6)
Use of un-prescribed injections in the last 3 months	93 (29.8)	27 (30.3)
Comorbidities (by self-report in the survey):		
Prior diagnosis of syphilis	111 (35.6)	35 (39.3)
Prior diagnosis of HIV	62 (24.1)	21 (27.6)
Not taking ART	34/62 (54.8)	11/21 (52.4)
Recent syphilis by laboratory testing	8/34 (23.5)	1/11 (9.1)
Diagnosis of STI in the last 6 months	22/34 (64.7)	5/11 (45.5)
Primary role during anal sex		
Insertive	87 (27.9)	2 (2.3)
Receptive	68 (21.8)	60 (67.4)
Versatile (e.g. both)	157 (50.3)	27 (30.3)
Stable^b^ partner, last 3 months	141 (45.2)	47 (52.8)
Sex with a male partner, last 3 months	312 (100)	81 (100)
Median number of male partner(s), last 3 months (IQR)	4 (2, 7)	15 (5, 40)
Sex with a female partner, last 3 months	45 (14.4)	1 (1.1)
Alcohol use disorder (AUDIT score > 8)	139 (44.6)	42 (47.2)
*Laboratory*		
Syphilis infection		
Recent syphilis (RPR ≥ 1:16)	52 (16.8)	6 (6.7)
Prior syphilis infection^c^ (RPR between 1:1–1:8)	79 (25.3)	45 (50.6)
No syphilis infection^d^	157 (50.8)	38 (42.7)
HIV infection	94 (30.1)	30 (33.7)
Total *Chlamydia trachomatis* (CT)	57 (18.6)	22 (24.7)
Anal CT	43 (14.1)	12 (13.5)
Pharyngeal CT	14 (4.5)	10 (11.2)
Total *Neisseria gonorrheae* (NG)	44 (14.2)	17 (19.1)
Anal NG	26 (8.4)	10 (11.2)
Pharyngeal NG	18 (5.8)	7 (7.9)
Co-infections with recent syphilis (*n* = 58, MSM [*N* = 52]; Transwomen [*N* = 6])		
HIV	23 (44.2)	4 (66.7)
Total CT	21 (40.4)	0 (0)
Anal CT	17 (32.7)	0 (0)
Pharyngeal CT	4 (7.7)	0 (0)
Total NG	10 (19.2)	1 (16.7)
Anal NG	6 (11.5)	1 (16.7)
Pharyngeal NG	4 (7.7)	0 (0)

Human immunodeficiency virus (HIV); antiretroviral therapy (ART); Rapid plasma reagin (RPR); *Treponema Pallidum* Particle Agglutination (TPPA); *Chlamydia trachomatis* (CT); *Neisseria gonorrheae* (NG); and sexually transmitted infection (STI)

^a^Living wage was approximately $250 USD per month in 2013–2014

^b^The participant self-identified that they had a stable sexual partner

^c^TPPA positive individuals that did not meet criteria for recently acquired syphilis infection

^d^RPR-negative and TPPA-negative individuals

Among our participants with a prior diagnosis of HIV, 54.8% of MSM and 52.4% of transwomen reported that they were not on ART (Table 1). Among HIV infected MSM not taking ART, 23.5% had recently acquired syphilis infection detected by laboratory testing and 64.7% had a diagnosis of a STI in the last 6 months. Among HIV infected transwomen participants not taking ART, 9.1% had recently acquired syphilis infection detected by laboratory testing and 45.5% had a diagnosis of a STI in the last 6 months.

Of the inclusion criteria needed for inclusion, the majority of the cohort reported over 5 years of sexual activity, more than five events of condomless anal sex in the past 6 months, and more than five sex partners in the past 3 months (Table 2).

**Table 2:** Frequency of inclusion criteria among men who have sex with men and male-to-female transgender participants of the PICASSO cohort, Lima, Peru, 2013–2014 (*n* = 401)

Inclusion Criteria	MSM	Transwomen
	n (*N* = 312) (%)	n (*N* = 89) (%)
MSM or transwomen	312 (100)	89 (100)
Age greater or equal to 18 years	312 (100)	89 (100)
Seeking care for HIV or STIs	312 (100)	89 (100)
Willing to return for eight follow up visits over 2 years	312 (100)	89 (100)
*Meet at least three of the following criteria:*		
1. More than 5 years of sexual activity	289 (92.6)	87 (98.0)
2. More than five occurrences of condomless anal sex in the past 6 months	225 (72.1)	73 (82.0)
3. More than five sex partners in the past 3 months	197 (63.1)	81 (91.0)
4. A positive syphilis test in the past 2 years	128 (41.0)	40 (45.0)
5. Any STI diagnosis within the past 6 months	145 (46.5)	22 (24.7)
6. Any syndromic ulcer-related STI at the time of screening	138 (44.2)	23 (25.8)
7. Diagnosed with HIV infection	62 (19.9)	24 (27.0)

Men who has sex with men (MSM); Transwomen (male-to-female transwomen); Human immunodeficiency virus (HIV); Sexually transmitted infection (STI)

### Prevalence of recently acquired syphilis infection and other STIs

Laboratory testing showed that the baseline prevalence of recently acquired syphilis infection was 16.8% for MSM and 6.7% for transwomen participants (Table 1). Among MSM, 30.1% were infected with HIV, 14.1% were infected with anal CT, 4.5% were infected with pharyngeal CT, 8.4% were infected with anal NG, and 5.8% were infected with pharyngeal NG. Among transwomen participants, 33.7% were infected with HIV, 13.5% were infected with anal CT, 11.2% were infected with pharyngeal CT, 11.2% were infected with anal NG, and 7.9% were infected with pharyngeal NG. Co-infection rates among the 52 MSM with recently acquired syphilis infection included: 44.2% with HIV, 40.4% with CT (32.7% was anal CT and 7.7% was pharyngeal CT), and 19.2% with NG (11.5% was anal NG and 7.7% was pharyngeal NG). Co-infection rates among the six transwomen participants with recently acquired syphilis infection included: 66.7% with HIV, 0% with CT, and 16.7% with NG (16.7% was anal NG).

### Characteristics associated with recently acquired syphilis infection

In multivariable analysis of pooled MSM and transwomen data, recently acquired syphilis infection was independently associated with younger age (adjusted prevalence ratio [aPR] = 0.96, 95% CI = 0.93–0.99; Table 3). Among behavioral characteristics, receptive role during anal sex (aPR = 2.56, 95% CI = 1.05–6.25) was independently associated with recently acquired syphilis infection. Among biological characteristics, self-reported prior HIV diagnosis (aPR = 1.70, 95% CI = 1.11–2.61), anal CT or NG infection (aPR = 1.69, 95% CI = 1.09–2.60), and self-reported prior syphilis diagnosis, which did not include infection detected during the study, (aPR = 3.53, 95% CI = 2.20–5.68) were independently associated with recently acquired syphilis infection. Latent class analysis did not identify any subgroups in our cohort with regard to our primary outcome variable, recently acquired syphilis infection.

**Table 3:** Associations between recently acquired syphilis infection and characteristics of PICASSO study participants, Lima, Peru, 2013–2014

Baseline characteristics	Recently acquired syphilis infection prevalence	Bivariate analysis	Multivariate analysis
	n/N (%)	Crude PR (95% CI)	Adjusted PR (95% CI)
Overall	58/285 (20.4)		
Age, years		**0.97 (0.94–0.99)**	**0.96 (0.93–0.99)**
18–25	22/98 (22.5)		
26–30	13/56 (23.2)		
31–35	13/50 (26.0)		
36+	10/81 (12.4)		
Number of recent male/transwomen sex partners (quartiles)			
0–2	21/86 (24.4)	Ref	
3–5	18/84 (21.4)	0.88 (0.50–1.53)	
6–10	8/50 (16.0)	0.66 (0.31–1.37)	
11+	11/65 (16.9)	0.69 (0.36–1.34)	
Role during anal sex			
Insertive	5/71 (7.0)	Ref	Ref
Receptive	22/85 (25.9)	**3.68 (1.46–9.22)**	**2.56 (1.05–6.25)**
Versatile	31/129 (24.0)	**3.41 (1.39–8.40)**	1.99 (0.82–4.85)
Condomless anal sex with a male partner, last 3 months			
No	14/70 (20.0)	Ref	
Yes	44/215 (20.5)	1.02 (0.59–1.75)	
Condomless anal sex with stable male partners, last 3 months			
No	21/97 (21.7)	Ref	
Yes	37/188 (19.7)	0.91 (0.56–1.46)	
Any anal sex partner met over the Internet in last 3 months			
No	34/182 (18.7)	Ref	
Yes	24/103 (23.3)	1.25 (0.78–1.98)	
Any anal sex with an anonymous sex partner in last 3 months			
No	30/140 (21.4)	Ref	
Yes	28/145 (19.3)	0.90 (0.57–1.43)	
Any anal sex in a sex club in last 3 months			
No	46/244 (18.9)	Ref	
Yes	12/41 (29.3)	1.55 (0.90–2.67)	
HIV infection			
Negative	31/206 (15.1)	Ref	Ref
Known infected^a^	20/50 (40.0)	**2.66 (1.66–4.25)**	**1.70 (1.11–2.61)**
Newly diagnosed	7/29 (24.1)	1.60 (0.78–3.31)	1.37 (0.72–2.60)
Any anal CT or NG infection			
Negative	38/229 (16.6)	Ref	Ref
Positive	20/56 (35.7)	**2.15 (1.36–3.40)**	**1.69 (1.09–2.60)**
Previous syphilis diagnosis (per patient interview)			
No	24/213 (11.3)	Ref	Ref
Yes	34/71 (47.9)	**4.25 (2.71–6.66)**	**3.53 (2.20–5.68)**
Alcohol use disorder (AUDIT score ≥ 8)			
No	32/151 (21.2)	Ref	
Yes	26/134 (19.4)	0.92 (0.58–1.45)	

Transwomen (male-to-female transwomen); Human immunodeficiency virus (HIV); *Chlamydia trachomatis* (CT); and *Neisseria gonorrheae* (NG)

^a^Self reported on baseline survey

^b^Bolded text indicates a *p*-value <0.05

## Discussion

The PICASSO cohort study successfully recruited 401 at risk participants, 312 MSM and 89 transwomen, with a high baseline prevalence of recently acquired syphilis infection in Lima, Peru. Among participants, the prevalence of recently acquired syphilis infection was 16.8% among MSM and 6.7% among tranwomen and HIV infection was 30.1% among MSM and 33.7% among transwomen participants. Among participants with recently acquired syphilis infection, STI and HIV co-infection were high. We found that recently acquired syphilis infection was independently associated with younger age, receptive anal sex, rectal bacterial STIs, self-reported prior HIV diagnosis, and prior syphilis diagnosis.

A limited number of studies report syphilis data in Peru. A study conducted prior to the introduction of ART showed a decrease in recently acquired syphilis infection (defined as a VDRL or RPR titer ≥1:16) from 8.6 in 1996 to 3.4% in 2002 [15]. Among MSM, two studies conducted after introduction of ART estimated the prevalence of recently acquired syphilis infection was 5.4 and 10.5% [16, 17]. Another study from 2012 reported that among HIV uninfected MSM and transwomen, 18% had positive serology for syphilis [18]. Prior studies differ from our cohort because we sampled at-risk MSM and transwomen recruited from HIV/STI clinics in the post-ART era. Our cohort was thoroughly surveyed and tested and was found to have a high incidence of recently acquired syphilis infection.

Prior reports described independent associations between recently acquired syphilis infection and HIV infection, increased number of years of sexual activity, and condomless anal intercourse among MSM and transwomen [16, 19]. We found that recently acquired syphilis infection was independently associated with rectal bacterial STIs and self-reported HIV diagnosis.

We found that among participants with a prior diagnosis of HIV, the majority of MSM and transwomen were not on ART. Among HIV-infected MSM and transwomen participants not taking ART, many participants had a recently acquired syphilis infection detected by laboratory testing and about half of all participants had a diagnosis of any STI in the last 6 months. Those data imply that HIV-infected MSM and transwomen not on ART had recent sex with increased risk of HIV infection transmission [20]. Improved management of HIV infection among MSM and transwomen is needed in Peru to prevent further spread of infection.

Our study has the following limitations. The clinic-based cohort was neither population-based nor randomly selected. We had limited statistical power to compare MSM versus transwomen in our study. Comparison analyses were conduced between MSM and transwomen in our cohort and showed no significant differences in identified risk factors. Additionally, latent class analysis was performed to ensure there were no identifiable subgroups in our cohort with regard to our primary outcome variable (i.e., recently acquired syphilis infection) [14]. The study’s inclusion criteria make it unlikely that participants were representative of MSM and transwomen in Lima, Peru.

## Conclusions

We successfully recruited a cohort of MSM and transwomen with a high prevalence of recently acquired syphilis infection in Lima, Peru. Recently acquired syphilis infection was associated with socio-demographic characteristics, sexual risk behavior, and sexually transmitted co-infections. Because HIV transmission and acquisition is associated with recently acquired syphilis infection, public health strategies need to be updated in Peru to recommend routine syphilis screening in HIV care for MSM and transwomen.

## Abbreviations

ART: Antiretroviral therapy
CI: Confidence interval
CT: *Chlamydia trachomatis*
HIV: Human immunodeficiency virus
MSM: Men who have sex with men
NG: *Neisseria gonorrheae*
PR: Prevalence ratio
RPR: Rapid plasma reagin
STI: Sexually transmitted infection
TPPA: *Treponema Pallidum* Particle Agglutination
Transwomen: Male-to-female transgender women
VDRL: Venereal Disease Research Laboratory
